# Intestinal pooling of taurine conjugated bile acids is associated with tissue aging and senescent immune evasion

**DOI:** 10.3389/fimmu.2026.1845117

**Published:** 2026-07-28

**Authors:** Jinzhuo Tan, Wenyan Xiong, Yingna Feng, Xingjie Li, Hongyu Jiang, Zongde Zhang

**Affiliations:** 1 Inflammation & Allergic Diseases Research Unit, Affiliated Hospital of Southwest Medical University, Luzhou, Sichuan, China; 2 Department of Clinical Laboratory, Chengdu Seventh People’s Hospital (Affiliated Cancer Hospital of Chengdu Medical College), Chengdu, Sichuan, China; 3 The School of Basic Medical Sciences, Southwest Medical University, Luzhou, Sichuan, China; 4 Department of Clinical Laboratory, Guangyuan Central Hospital, Guangyuan, Sichuan, China

**Keywords:** aging, gut, immune evasion, PD-L1, RARa, taurine-conjugated bile acids

## Abstract

**Background:**

Aging drives metabolic decline and the accumulation of senescent cells that evade immune clearance. The mechanisms linking host-microbiome co-metabolism to peripheral tissue deterioration remain unclear. We hypothesized that age-dysregulated enterohepatic bile acid (BA) signaling directly promotes systemic aging and immune senescence.

**Methods:**

We conducted targeted BA metabolomics in the intestinal and peripheral compartments of young and middle-aged mice. To establish causality, 12-month-old mice underwent systemic BA depletion via a cholestyramine diet, followed by assessments of Farnesoid X Receptor (FXR) and Retinoic Acid Receptor alpha (RARα) signaling, cellular senescence, PD-L1/PD-L2 expression, and multi-organ transcriptomics.

**Results:**

Advancing age sequesters potent endogenous FXR antagonists (T-α-MCA, T-ω-MCA) within the gut lumen. However, peripheral tissues (lungs, skin, lymph nodes) progressively decrease in the levels of these regulatory molecules. This spatial disconnect triggers chronic peripheral FXR hyperactivation, disrupting local RARα signaling. which accelerates the accumulation of senescent cells and actively shields them from immune clearance by upregulating PD-L1 and PD-L2 surface checkpoints. Crucially, systemic BA sequestration reversed this phenotype. Depleting the pathological BA pool suppressed peripheral FXR overreaction. Further cleared the senescent burden, stripped senescent cells of their immune-evasive checkpoints, and drove a multi-organ of age-associated transcriptional changes.

**Conclusion:**

The age-altered BA metabolome may contribute to systemic aging and to upregulation of PD-L1 and PD-L2. Rebalancing the enterohepatic BA network to relieve peripheral FXR hyperactivation represents a potent, broad-spectrum geroprotective strategy to restore immune surveillance and multi-organ homeostasis.

## 1 Introduction

Aging is a complex biological process characterized by the progressive decline in tissue and organ function, which increases susceptibility to numerous diseases (1–3). A key driver of this process is the accumulation of senescent cells that have entered an irreversible state of growth arrest in various tissues (4). Two major signaling pathways initiate and maintain this growth arrest: the p53-p21-retinoblastoma protein (RB) pathway and the p16INK4A-RB pathway (5). These cells are not inert; they secrete a pro-inflammatory cocktail of factors, known as the senescence-associated secretory phenotype (SASP), which disrupts tissue homeostasis, promotes chronic inflammation, and contributes to the manifestation of age-related pathologies (5, 6). While cell-autonomous mechanisms driving senescence, such as DNA damage and telomere attrition (7), are well documented, the systemic, non-cellular factors that regulate the accumulation of senescent cells throughout the organism remain less well understood.

Among the systemic factors that change with age are endogenous metabolites, including bile acids (BAs) (8). Primarily known for their role in facilitating lipid digestion and absorption, BAs also function as potent signaling molecules that regulate a wide array of metabolic and inflammatory pathways (9). This signaling is predominantly mediated by the nuclear Farnesoid X Receptor (FXR) (10). The composition of the circulating BA pool is heavily influenced by the gut microbiome (11–13), which performs critical modifications to primary BAs synthesized in the liver. Activation of the nuclear receptor FXR in the ileum serves as negative feedback control for BA synthesis (14). Given that the gut microbiome undergoes significant alterations with age, it is plausible that age-related dysbiosis dysregulates BA signaling, which, in turn, could influence systemic aging.

Recent evidence has begun to link BA signaling to pathways involved in cellular fate. Notably, BAs have been shown to activate retinoic acid signaling by directly interacting with the Retinoic Acid Receptor α (RARα) and the FXR (15, 16), a pathway implicated in cell cycle control (17, 18). Furthermore, the expression levels of both FXR and RARα have been observed to change in various tissues during the aging process (19–22). However, it remains unclear whether systemic dysregulation of the BA-FXR-RARα signaling axis is a causal factor in the accumulation of senescent cells and the progression of organismal aging.

Here, we investigate the hypothesis that an age-associated, tissue-specific dysregulation of BA profiles promotes senescence by activating the FXR-RARα signaling pathway in peripheral tissues. We characterized the BA landscape across multiple tissues in young and middle-aged mice, revealing a striking dichotomy that suggests peripheral FXR over-activation. Systemically depleting BAs in middle-aged mice reverses key features of aging by inhibiting the FXR-RARα axis, ameliorating cellular senescence, and upregulating youthful gene expression profiles across multiple organ systems. This work identifies dysregulated bile acid signaling as a key driver of aging and proposes therapeutic modulation of this pathway as a promising strategy to promote health span.

## 2 Materials and methods

### 2.1 Mice

C57BL/6J (11 months old) mice were purchased from Chengdu Yaokang Biotechnology. The 12–15 month timepoint was selected to capture early aging-associated metabolic alterations prior to severe late-life frailty. All mice were bred and maintained at Southwest Medical University under specific pathogen-free conditions.

### 2.2 Mice models

The C57BL/6J (11 months old) mice were fed with 4% cholestyramine for 4 weeks. Control mice were treated with a regular diet only. On day 29, mice were sacrificed for sample collection.

### 2.3 Reagents

The following reagents were purchased: SPiDER-β-Gal (DOJINDO); cholestyramine (Sigma-Aldrich).

### 2.4 FACS and antibody

Lung, liver, kidney, spleen, mLN, and iLN cells were incubated with the SPiDER-β-Gal (1 μmol/L) in serum-free Hank’s buffer at 37 °C for 15 min, then washed three times with Hank’s buffer. Next, these cells were incubated with fluorochrome-coupled agents for 30 min at 4 °C, then washed and suspended in FACS buffer (2% FBS in PBS). Data were obtained using the Acea NovoCyte series flow cytometer. The following anti-murine antibodies were used for flow cytometry: PD-L1 (B7-H1) and PD-L2 (B7-DC) (all purchased from BioLegend).

### 2.5 Quantitative real-time PCR

gonadal white adipose tissue and inguinal white adipose tissue were ground into single cells. Total RNA was extracted using the Total RNA Isolation Kit (Vazyme). And cDNA was synthesized with a cDNA Synthesis Kit (Vazyme). Real-time PCR was performed on LightCycler480 (Roche) using SYBR Green Master Mix (Vazyme). Gene expression was normalized to GAPDH and calculated using the ΔΔCt method. PCR primers of mouse genes were purchased from GENERAY. GAPDH was used as an internal control.

### 2.6 Western blot

gWAT and skin were collected and lysed on ice with RIPA Buffer (Beyotime) containing protease/phosphatase inhibitor cocktail (Solarbio) and 1 mM PMSF (Solarbio). Cell extracts were loaded onto BiofurawTM Precast Gel (Beyotime), separated by electrophoresis, and transferred onto NC membranes (Millipore). Signals were generated with High-sig ECL Western Blotting Substrate (Beyotime). The antibodies: RARa rabbit mAb (A19551, ABclonal), Phospho-RARa (Ser77) rabbit mAb (P45-99142, Invitrogen), Phospho-RARa (Ser96) rabbit mAb (P45-64622, Invitrogen), FXR/NR1H4 mouse mAb (E4B8P, CST), GAPDH rabbit mAb (14C10, CST), P16INK4a rabbit pAb (A0262, Abclonal), P21CIP1 rabbit mAb (A19094, Abclonal), P53 mouse mAb (A10610, Abclonal). Band signals were quantified in ImageJ and analyzed in GraphPad Prism.

### 2.7 Immunofluorescence staining

Skins were carefully isolated, embedded in optimal cutting temperature compound, and snap-frozen in liquid nitrogen. Cryosections 12 μm thick were fixed for 30 min at 4 °C with 4% PFA. Then incubated with the following primary antibodies: RARa rabbit mAb (A19551, ABclonal), Phospho-RARA (Ser77) rabbit mAb (P45-99142, Invitrogen), P16INK4a rabbit pAb (A0262, Abclonal); Secondary antibody: FITC goat anti-rabbit IgG (A5011, Abclonal), Alexa Fluor 594-conjugated goat anti-rabbit IgG(A5039, Abclonal). DAPI (4′,6-diamidine-2-phenyl indole) staining of the nucleus. Images on an Olympus FV1000 microscope equipped with constant exposure.

### 2.8 UPLC-MS analysis of BAs

Skin, lungs, mLN, duodenum, and spleen tissues were collected. The BAs data acquisition instrument system mainly includes UPLC (ExionLC™ AD) and MS/MS (QTRAP^®^6500+). The analytical conditions were as follows: HPLC column, waters ACQUITY UPLC HSS T3 C18 (1.8 µm, 100 mm×2.1 mm i.d.) solvent system, ultrapure water (containing 0.01% acetic acid and 5mmol/L ammonium acetate), and acetonitrile (containing 0.01% acetic acid); flow rate, 0.35 ml/min; temperature, 40 °C; and injection volume: 3 μl; gradient program, 95:5 V/V at 0 min, 60:40V/V at 0.5 min, 50:50V/V at 4.5min, 25:75V/V at 7.5 min, 5:95V/V at 10min, 95:5V/V at 12min; The effluent was alternately connected to an ESI-triple quadrupole-linear ion trap (QTRAP)-MS. The UPLC-MS datasets for hierarchical clustering were processed in R.

### 2.9 RNA sequencing and analysis

Lungs, duodenum, skin, mLN, and spleen tissues were collected from mice, and the products were subjected to RNA isolation, library construction, and sequencing analysis by Novogene Technology Co., Ltd. (Beijing, China). RNA integrity was assessed using Agilent Bioanalyzer (RIN > 8.0). Libraries were prepared using the NEBNext Ultra II RNA Library Prep Kit and sequenced on Illumina NovaSeq 6000 (paired-end, 150 bp, depth > 30 million reads per sample). Reads were aligned to the mm10 genome using STAR (v2.7). FeatureCounts was used for quantification. DESeq2 (v1.32) was used for differential expression analysis with Benjamini-Hochberg FDR correction (q < 0.05).”

### 2.10 Statistical analysis

The statistical analysis was performed using GraphPad Prism. All plot graphs show means with standard deviation (SD); for comparisons involving more than two groups, a one-way ANOVA with Tukey’s HSD *post hoc* test was used. For two-group comparisons, a two-tailed Student’s t-test was used. A p-value of less than 0.05 was considered statistically significant. *p<0.05, **p<0.01, ***p< 0.001, not significant (N.S.).

## 3 Results

### 3.1 Age-associated spatiotemporal dysregulation of the BAs

**Figure 1:**
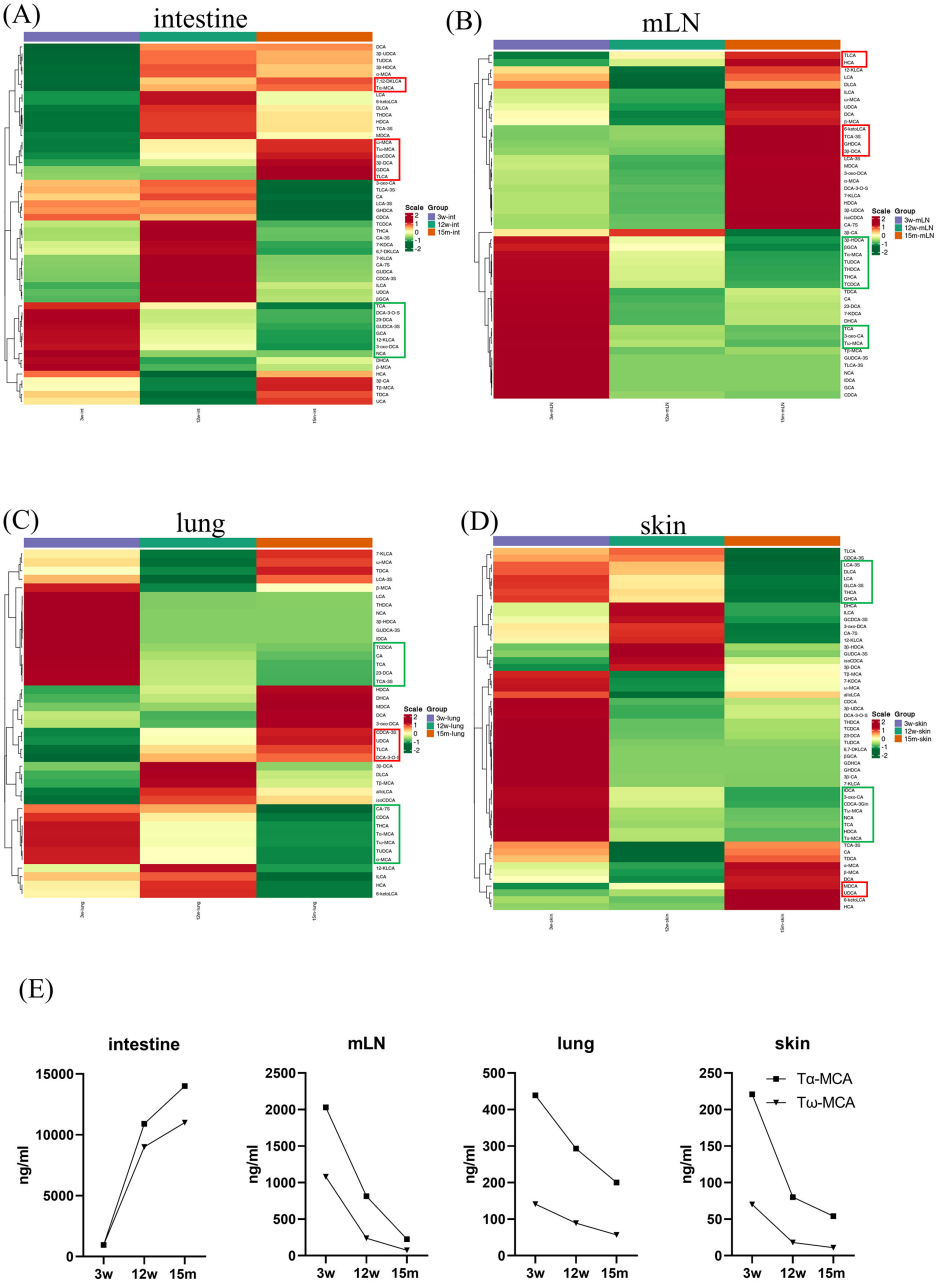
Age-associated dysregulation of bile acid profiles in murine tissues. **(A–D)** Heatmaps showing the relative abundance of bile acid (BA) species in the **(A)** intestinal contents, **(B)** mesenteric lymph nodes (mLN), **(C)** lung, and **(D)** skin of mice at 3 weeks (3w), 12 weeks (12w), and 15 months (15m) of age. Each row represents a specific BA, and each column represents an individual mouse sample. The color scale indicates relative abundance, with the red box representing higher levels and the green box representing lower levels. **(E)** Line graphs showing the quantification (ng/ml) of the key Farnesoid X Receptor (FXR) antagonist bile acids—tauro-α-muricholic acid (Tα-MCA), and tauro-ω-muricholic acid (T ω -MCA), across the three age groups in the intestine, mLN, lung, and skin. Data show a progressive increase in these BAs in the intestine with age, and a corresponding decrease in peripheral tissues.

To investigate the systemic dynamics of bile acid (BA) metabolism during aging, we performed targeted metabolomic profiling across distinct anatomical compartments. Tissues encompassing the intestinal lumen (int), a secondary lymphoid organ (mesenteric lymph node; mLN), and key barrier tissues (lung and skin) were harvested from mice at weaning (3 weeks; 3w), young adulthood (12 weeks), and middle-aged (15 months; 15m). Unsupervised hierarchical clustering of relative BA abundances revealed a profound, age-dependent restructuring of the systemic BA metabolome (**Figures 1A–D**). To systematically characterize this metabolic transition, we identified specific clusters of BAs that were significantly upregulated during aging (highlighted in red boxes) and those that were concurrently downregulated (highlighted in green boxes).

In the intestinal compartment, advancing age precipitated a massive accumulation of specific BA species. The upregulated BAs in the 15-month-old intestine (**Figure 1A**, red boxes) formed distinct clusters of highly enriched metabolites. Upon detailed inspection of these aging-associated shifts, we found that tauro-α-muricholic acid (Tα-MCA) and tauro-ω-muricholic acid (Tω-MCA) were the most prominently abundant bile acids that strictly increased in the aging intestine while simultaneously decreasing in all examined peripheral organs. Quantitative targeted analysis confirmed this profound localized pooling; the absolute concentrations of Tα-MCA and Tω-MCA surged dramatically from their baseline at 3 weeks to peak at 15 months within the intestinal lumen (**Figure 1E**, intestine).

In striking contrast to the intestinal compartment, the peripheral barrier tissues and immune organs experienced a precipitous, age-dependent decline in these same metabolites. Heatmap profiles of the mLN, lung, and skin demonstrated a youthful (3w) environment highly enriched with Tα-MCA and Tω-MCA, which subsequently formed the core of the downregulated BA clusters during aging (**Figures 1B–D**, green boxes). Quantitative validation confirmed that the high early-life concentrations of these specific BAs plummeted by 12 weeks and reached their absolute nadir in the 15-month-old cohort across all peripheral sites (**Figure 1E**).

Tα-MCA and Tω-MCA are potent endogenous FXR antagonists. This spatiotemporal disconnect reveals severe dysregulation of the aging enterohepatic axis. The concurrent, massive pooling of these FXR antagonists within the intestinal contents and their simultaneous depletion from the mLN, lung, and skin establishes an inverted systemic agonist-to-antagonist ratio. Collectively, these data support the hypothesis that the aging gut acts as a trap for endogenous FXR antagonists, progressively starving the periphery of these regulatory molecules, thereby driving a chronic FXR overreaction in peripheral tissues (skin, lung, mLN), ultimately driving the aging process.

### 3.2 Systemic bile acid depletion rescues age-induced FXR hyperactivation and restores the peripheral RARα signaling axis

**Figure 2:**
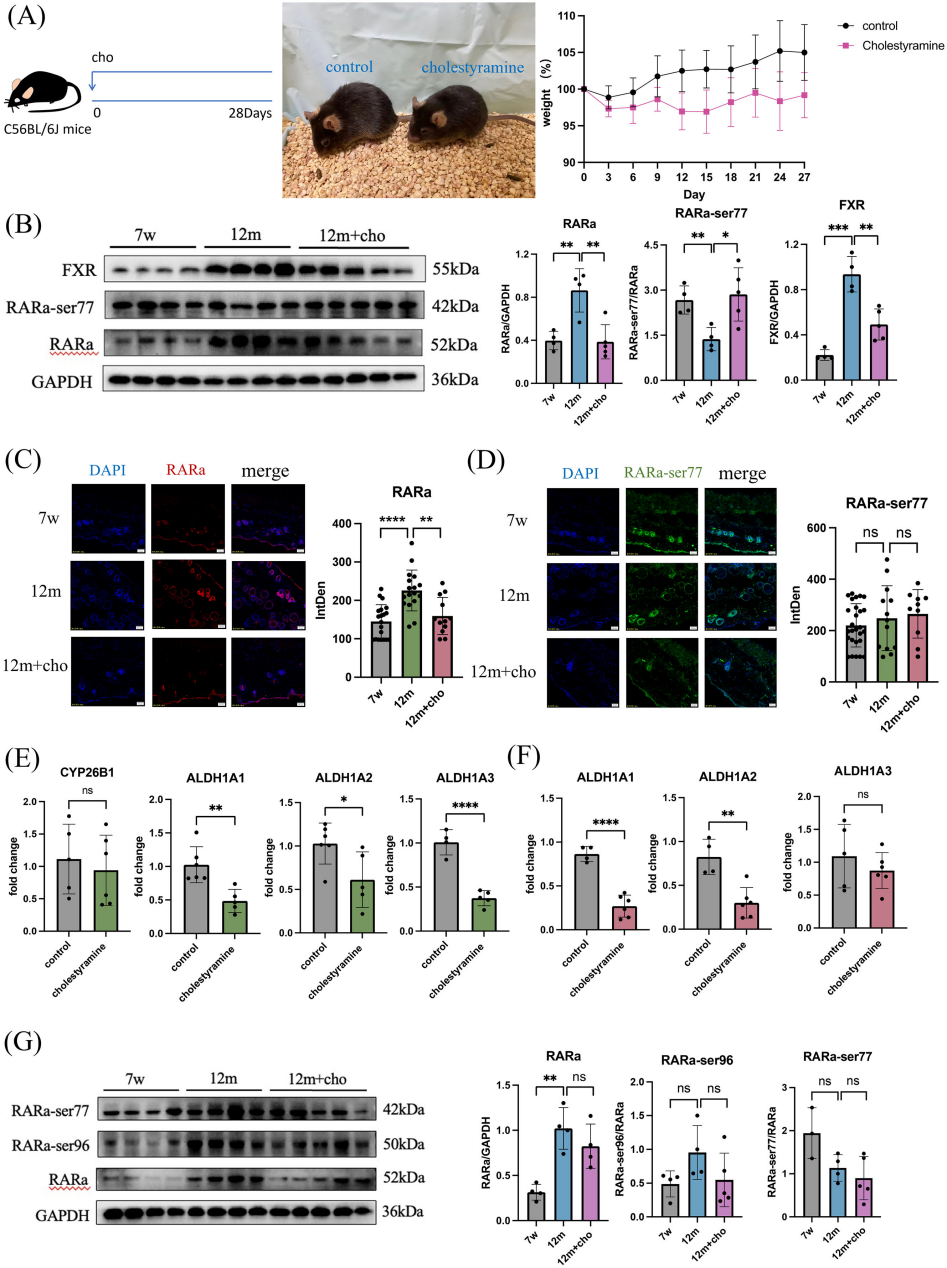
Bile acid depletion inhibits the FXR-RARα signaling axis in middle-aged mice. **(A)** Schematic of the experimental design. Wild-type C57BL/6J mice (12 months, male, n=6) were fed 4% cholestyramine or a regular diet. Four weeks later, mice were sacrificed—representative images and the weight records of the 12-month-old mice. **(B)** FXR, RARa, and RARa-ser77 expression were analyzed by western blot in skin. **(C)** Skins were stained with DAPI(blue) and RARa(red) antibody and analyzed by confocal imaging. The scale bars are 50μm. Quantification was performed using ImageJ software. **(D)** Skins were stained with DAPI(blue) and RARa-ser77(green) antibody and analyzed by confocal imaging. The scale bars are 50 μm. Quantification was performed using ImageJ software. **(E)** CYP26B1, ALDH1A1, ALDH1A2, and ALDH1A3 mRNA expression levels were determined by RT-qPCR in gWAT. **(F)** RT-qPCR determined ALDH1A1, ALDH1A2, and ALDH1A3 mRNA expression levels in iWAT. **(G)** RARa and RARa-ser77 or RARa-ser96 expression were analyzed by western blot in gWAT. Data are presented as mean ± SD; p-values were calculated using the two-tailed Student’s t-test. For comparisons involving more than two groups, a one-way ANOVA with Tukey’s HSD *post hoc* test was used. *p < 0.05, **p < 0.01, ***p < 0.001, ****p < 0.0001, ns: not significant.

To directly test the hypothesis that the age-associated pooling of endogenous FXR antagonists in the gut drives pathological FXR overreaction in the periphery, we employed a systemic bile acid (BA) depletion strategy. Wild-type middle-aged mice (12m) were fed a diet containing 4% cholestyramine, a BA sequestrant that interrupts the enterohepatic circulation, for 28 days (**Figure 2A**).

We first evaluated protein expression in the skin to confirm the peripheral overreaction phenotype. Consistent with the age-associated loss of local FXR antagonists observed in our metabolomic profiling, 12-month-old mice exhibited a profound and significant upregulation of total FXR protein compared to youthful (7-week-old) controls (**Figure 2B**). This hyperactivation was accompanied by a significant abnormal accumulation of total Retinoic Acid Receptor alpha (RARα), a critical regulatory network heavily interconnected with peripheral tissue homeostasis, as demonstrated by both Western blot (**Figure 2B**) and confocal immunofluorescence imaging (**Figure 2C**). Strikingly, depleting the systemic BA pool with cholestyramine (12m+cho) potently suppressed this hyperactivation, significantly reducing both skin FXR and total RARα expression back toward youthful baseline levels.

While total RARα was highly elevated in the middle-aged skin, the relative phosphorylation at serine 77 (RARα-ser77/RARα ratio) was significantly suppressed compared to 7-week-old mice (**Figure 2B**). Although the absolute intensity of RARα-ser77 fluorescence remained stable across the groups (**Figure 2D**), the massive accumulation of the total unphosphorylated protein pool effectively collapsed the proportional signaling balance. Cholestyramine treatment completely rescued this functional deficit, normalizing the total protein pool and restoring the RARα-ser77/RARα ratio to the levels observed in youth.

To determine whether this BA-driven axis extends beyond the skin into other peripheral metabolic compartments, we assessed white adipose tissue (WAT). RT-qPCR analysis demonstrated that systemic BA depletion significantly downregulated the mRNA expression of key retinoic acid-synthesizing enzymes—specifically ALDH1A1, ALDH1A2, and ALDH1A3 in gonadal WAT (gWAT) (**Figure 2E**), and ALDH1A1 and ALDH1A2 in inguinal WAT (iWAT) (**Figure 2F**). While aging similarly upregulated total RARα protein in gWAT (**Figure 2G**), the specific serine 77 and serine 96 phosphorylation ratios in this adipose depot were not significantly rescued by the 28-day cholestyramine intervention, suggesting tissue-specific sensitivities to the BA-FXR-RARα signaling cascade.

Collectively, these *in vivo* depletion experiments validate our hypothesis. The age-associated dysregulation of the BA pool directly drives a pathological FXR overreaction in peripheral tissues. Furthermore, suppressing this systemic BA-FXR axis is sufficient to restore the downstream RARα signaling balance in the skin, linking the altered intestinal metabolome to localized tissue-aging pathways.

### 3.3 Systemic bile acid depletion ameliorates cellular senescence in peripheral tissues

Having demonstrated that systemic bile acid (BA) depletion successfully rescues the pathological FXR overreaction and restores the balance of RARα signaling in the aged periphery, we next sought to determine whether this molecular restoration translates into an amelioration of the physical aging phenotype. Specifically, we evaluated the burden of cellular senescence, a primary driver of age-related tissue dysfunction, in the peripheral tissues of our middle-aged (12m) cohort following the 28-day cholestyramine (cho) intervention.

**Figure 3:**
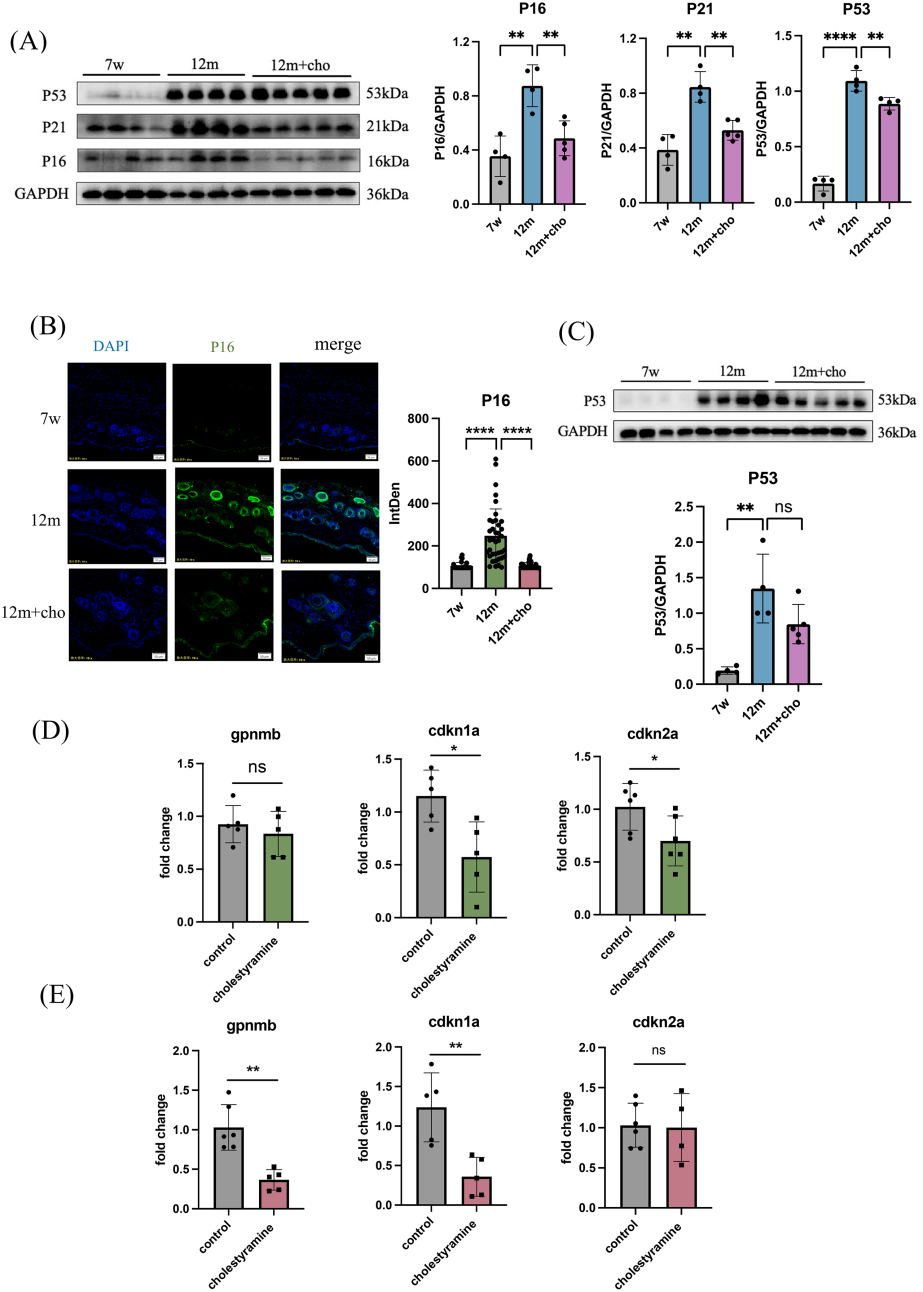
Bile acid depletion ameliorates cellular senescence in peripheral tissues. **(A)** P16, P21, and P53 expression was detected by Western blot in skin. **(B)** Skins were stained with DAPI(blue) and P16(green) antibody and analyzed by confocal imaging. The scale bars are 50 μm. Quantification was performed using ImageJ software. **(C)** P53 expression was analyzed by Western blot in gWAT. **(D)** *Gpnmb*, C*dkn1a* (p21) and *Cdkn2a* (p16) mRNA expression level were determined by RT-qPCR in gWAT **(E)** RT-qPCR determined *Gpnmb*, C*dkn1a* (p21), and *Cdkn2a* (p16) mRNA expression levels in iWAT. Data are presented as mean ± SD; p-values were calculated using the two-tailed Student’s t-test. For comparisons involving more than two groups, a one-way ANOVA with Tukey’s HSD *post hoc* test was used. *p < 0.05, **p < 0.01, ***p < 0.001, ****p < 0.0001, ns: not significant.

We first assessed the expression of classic senescence-associated proteins, including p53/p21 and p16 in the tumor suppressor pathways, and Gpnmb in the skin (23–26). Consistent with advanced chronological age, 12m control mice exhibited a profound and significant accumulation of the core senescence regulators P16, P21, and P53 compared to youthful 7-week-old (7w) mice, as determined by Western blot analysis (**Figure 3A**). Strikingly, interrupting the enterohepatic circulation with cholestyramine (12m+cho) potently reversed this senescent signature, significantly downregulating the protein expression of P16, P21, and P53 back toward the baseline levels observed in youth. This dramatic reversal was further corroborated by confocal immunofluorescence imaging of the skin, which visually confirmed the massive age-induced accumulation of P16 in the 12m cohort and its subsequent, statistically significant clearance following systemic BA depletion (**Figure 3B**).

To determine whether this anti-senescence effect was conserved across other peripheral metabolic compartments, we evaluated senescence burden in white adipose tissue (WAT). In gonadal WAT (gWAT), aging similarly drove a significant upregulation of total P53 protein (**Figure 3C**). While the cholestyramine-induced reduction of P53 protein in gWAT trended downward without reaching statistical significance, transcriptomic analysis revealed targeted functional rescue. RT-qPCR quantification demonstrated that systemic BA depletion significantly downregulated the mRNA expression of key senescence markers cdkn1a (p21) and cdkn2a (p16) in gWAT, with the senescence-associated marker gpnmb trending downward (**Figure 3D**).

A similar pattern of targeted senescence amelioration was observed in inguinal WAT (iWAT). Cholestyramine treatment significantly suppressed the mRNA expression of gpnmb and cdkn1a (p21) compared to the middle-aged controls, although the expression of cdkn2a (p16) remained unaffected in this specific depot (**Figure 3E**).

Collectively, these data reveal that the pathological pooling of endogenous FXR antagonists in the aged gut is not merely an isolated metabolic phenomenon, but a direct upstream driver of peripheral tissue aging. By depleting the systemic BA pool and suppressing the consequent peripheral FXR overreaction, we successfully ameliorated the molecular hallmarks of cellular senescence across multiple peripheral tissues, functionally linking the intestinal metabolome to systemic aging.

### 3.4 Systemic bile acid depletion suppresses immune checkpoint expression on peripheral senescent cells

We next investigated the functional consequences of this intervention on senescent cell immunogenicity. A critical mechanism by which senescent cells persist and accumulate in aged tissues is through the evasion of immune clearance, often achieved by upregulating surface immune checkpoint molecules such as Programmed Death-Ligand 1 (PD-L1) (27) and Programmed Death-Ligand 2 (PD-L2) (28). Therefore, we sought to determine if our BA depletion strategy altered the immune-evasive profile of the senescent cell population in aged mice.

**Figure 4:**
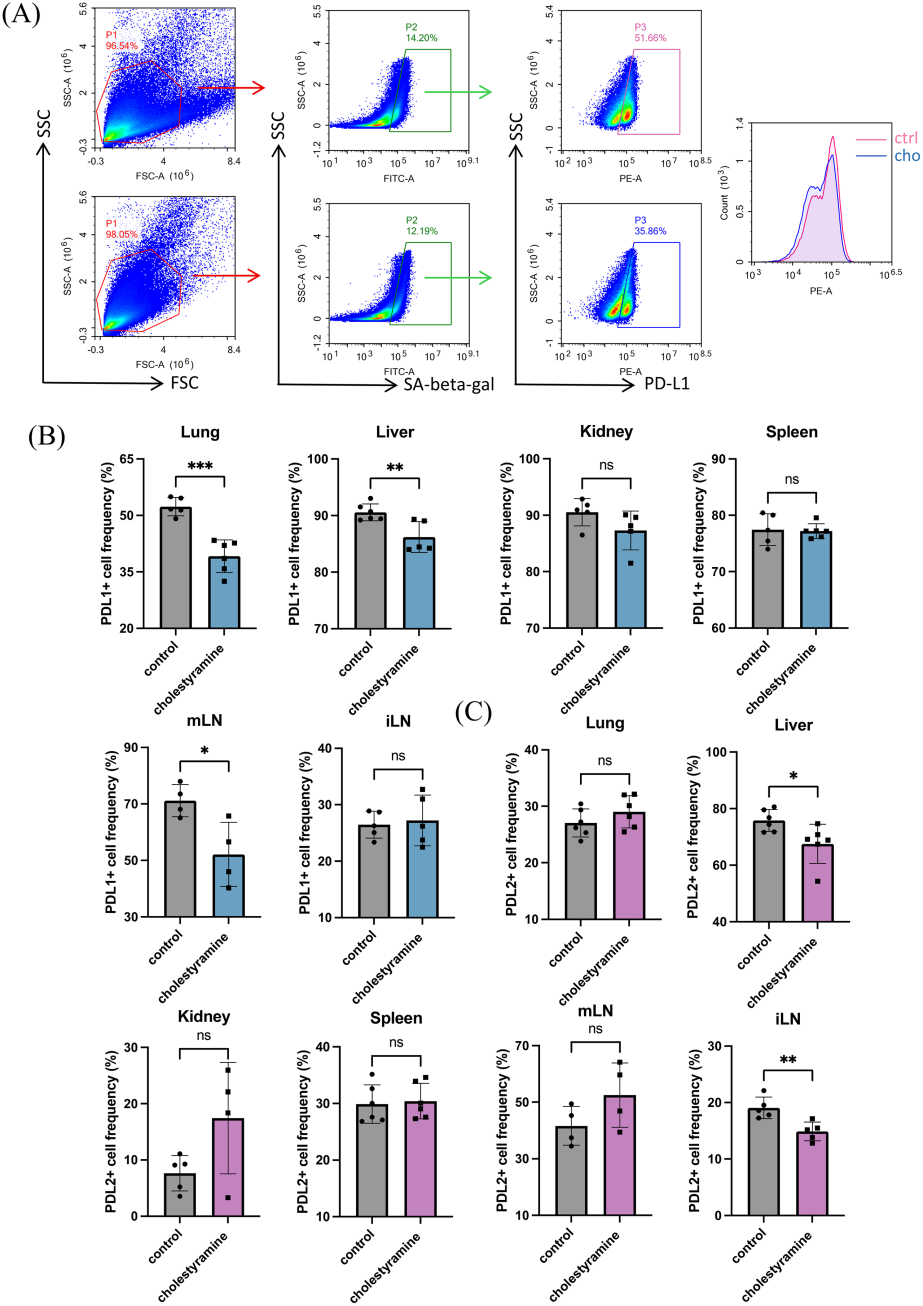
Bile acid depletion reduces PD-L1 and PD-L2 expression on senescent cells. **(A)** PD-L1 and PD-L2 fluorescent signals were analyzed by flow cytometry in senescent cells(SPiDER-β-gal+cells) from the lung, as shown in the gating strategy. **(B)** Wild-type C57BL/6J mice (12 months, male, n=6) were fed 4% cholestyramine for 4weeks. PD-L1+ senescent cells (SPiDER-β-gal+ cells) were analyzed by flow cytometry for frequency in the lung, liver, kidney, spleen, mLN, and iLN. **(C)** Wild-type C57BL/6J mice (12 months, male, n=6) were fed 4% cholestyramine for 4weeks. PD-L2+ senescent cells (SPIDER-β-gal+ cells) were detected by flow cytometry in the lung, liver, kidney, spleen, mLN, and iLN. Data are presented as mean ± SD; p-values were calculated using the two-tailed Student’s t-test. For comparisons involving more than two groups, a one-way ANOVA with Tukey’s HSD *post hoc* test was used. *p < 0.05, **p < 0.01, ***p < 0.001, ****p < 0.0001, n.s: not significant.

We utilized flow cytometry to specifically identify senescent cells across multiple peripheral organs in our 12-month-old (12m) cohort by gating on the lysosomal hydrolase senescence-associated β-galactosidase (SA-β-GAL, SPiDER-β-gal+) positivity (**Figure 4A**), a key senescent cell marker detected at the tissue level (29). Within this specific senescent population, we then quantified the frequency of cells expressing the inhibitory checkpoints PD-L1 and PD-L2 following the 4-week cholestyramine (cho) intervention.

Analysis of the PD-L1 expression profile revealed a profound, tissue-specific suppression following systemic BA depletion. In control 12m mice, a substantial proportion of the senescent cells expressed PD-L1. However, interrupting the enterohepatic circulation with cholestyramine significantly reduced the frequency of PD-L1+ senescent cells in critical barrier, metabolic, and mucosal immune tissues—specifically the lung, liver, and mesenteric lymph node (mLN) (**Figure 4B**). While the kidney, spleen, and inguinal lymph node (iLN) did not exhibit statistically significant reductions, the robust suppression in the lung, liver, and mLN indicates a major shift in the immunogenic profile of senescent cells residing in tissues highly exposed to circulating metabolites and gut-derived signals.

We subsequently evaluated PD-L2 expression in the same SPiDER-*β*-gal+ senescent populations. While the systemic impact on PD-L2 was more limited than that on PD-L1, cholestyramine treatment still induced a significant reduction in the frequency of PD-L2+ senescent cells in the liver and the iLN (**Figure 4C**). Expression levels in the lung, kidney, spleen, and mLN remained largely unchanged, suggesting that the BA-driven modulation of immune checkpoints exhibits distinct, tissue-specific regulatory patterns.

Collectively, these flow cytometry data suggest that the pathological BA pool in the aged host not only drives the initial development of cellular senescence but also actively maintains this senescent burden by promoting an immune-evasive PD-L1/PD-L2 phenotype. By sequestering the systemic BA pool, we successfully stripped the remaining senescent cells of these inhibitory checkpoint shields in key peripheral tissues, potentially restoring their susceptibility to immune-mediated clearance and further reinforcing the link between the intestinal metabolome and peripheral tissue homeostasis.

### 3.5 Systemic bile acid depletion drives transcriptomic changes and restores tissue-specific structural and metabolic integrity

To comprehensively evaluate the organism-wide impact of the pathological bile acid (BA) pool and determine whether our intervention restored global tissue function, we performed bulk RNA sequencing (RNA-seq) across diverse peripheral and mucosal compartments. By comparing the transcriptomes of youthful (7w), middle-aged (12m), and BA-depleted middle-aged (12m+cho) mice, we systematically mapped the systemic gene expression networks responsive to the aging enterohepatic axis.

**Figure 5:**
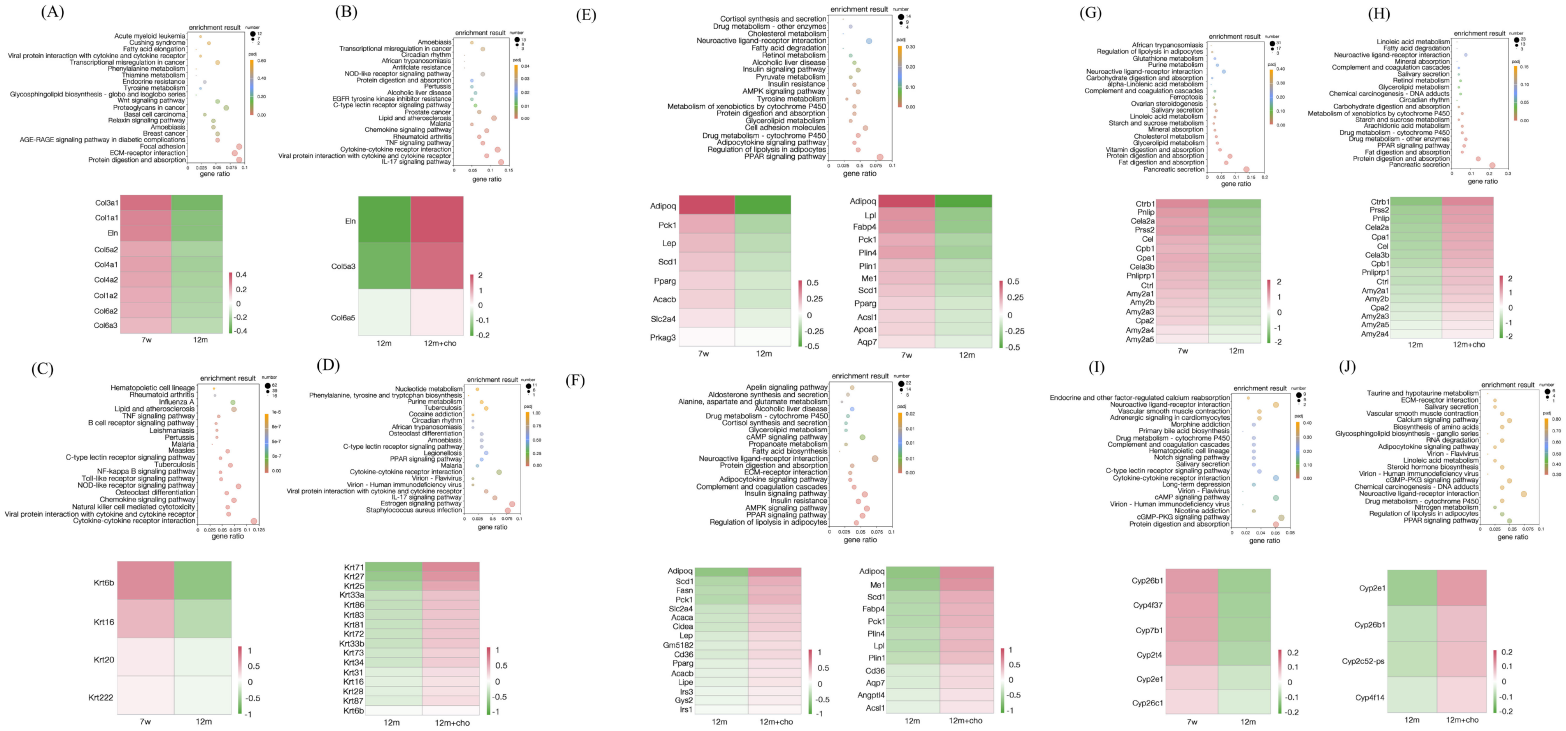
Transcriptomic analysis reveals a systemic reversal of age-related gene expression by bile acid depletion. KEGG pathway enrichment analysis and corresponding heatmaps of differentially expressed genes from RNA sequencing of various tissues from young (7w), middle-aged (12m), and middle-aged cholestyramine-treated (12m+cho) mice. Bubble plots show significantly enriched pathways, with gene ratio on the x-axis and color/size indicating statistical significance and number of genes, respectively. Heatmaps show the relative expression of selected genes, with red indicating higher expression and green/white indicating lower expression. **(A)** Downregulated pathways and associated collagen/elastin genes in the lung of 12m vs. 7w mice. **(B)** Upregulated pathways and restored expression of collagen/elastin genes in the lung of 12m+cho vs. 12m mice. **(C)** Downregulated pathways and associated keratin genes in the skin of 12m vs. 7w mice. **(D)** Upregulated pathways and restored expression of keratin genes in the skin of 12m+cho vs. 12m mice. **(E)** Downregulated pathways and associated genes in the AMPK, PPAR, and CYP450 pathways in the mesenteric lymph nodes (mLN) of 12m vs. 7w mice. **(F)** Upregulated pathways and restored expression of genes in the AMPK, PPAR, and CYP450 pathways in the mLN of 12m+cho vs. 12m mice. **(G)** Downregulated pathways and associated genes related to digestion and metabolism in the duodenum of 12m vs. 7w mice. **(H)** Upregulated pathways and restored expression of digestion and metabolism-related genes in the duodenum of 12m+cho vs. 12m mice. **(I)** Downregulated pathways and associated CYP450 family genes in the spleen of 12m vs. 7w mice. **(J)** Upregulated pathways and restored expression of CYP450 family genes in the spleen of 12m+cho vs. 12m mice.

Pathway enrichment analysis revealed that advancing chronological age (12m) precipitated a profound, systemic transcriptional suppression of fundamental tissue-specific identities. In the lung, aged mice exhibited a significant downregulation of pathways governing extracellular matrix (ECM)-receptor interactions (**Figure 5A**). Upon detailed inspection, this structural decline was driven by the severe transcriptional loss of critical barrier components, notably collagens (*Col3a1*, *Col1a1*) and elastin (*Eln*). Strikingly, depleting the systemic BA pool with cholestyramine (12m+cho) completely reversed this age-associated decline, robustly upregulating these identical networks and restoring the transcript pool of key matrix genes (**Figure 5B**). A parallel rescue occurred in the cutaneous barrier. The 12m skin suffered a massive downregulation of keratinocyte differentiation networks (**Figure 5C**), which was potently reversed following systemic BA sequestration. The intervention drove the comprehensive re-expression of a vast array of suppressed keratin genes, including *Krt71*, *Krt27*, and *Krt16*, restoring the foundational transcriptional architecture of the skin (**Figure 5D**).

Beyond physical barriers, the dysregulated aged BA pool profoundly suppressed critical metabolic and regulatory nodes within peripheral immune organs and the gastrointestinal tract. In the mesenteric lymph node (mLN), aging severely silenced primary metabolic hubs, most notably the AMPK and PPAR signaling pathways, leading to the targeted downregulation of essential lipid and energy metabolism genes (*Adipoq*, *Pparg*, *Scd1*) (**Figure 5E**). Systemic BA sequestration successfully reactivated these pathways in aged mLN, driving robust re-expression of these foundational metabolic regulators (**Figure 5F**). Similarly, the widespread age-induced silencing of Cytochrome P450 (CYP450) family genes in the spleen (*Cyp26b1*, *Cyp2e1*) (**Figure 5I**) was potently reversed following cholestyramine treatment (**Figure 5J**). Finally, within the local gastrointestinal tract (duodenum), the severe age-associated downregulation of pathways governing macronutrient digestion and pancreatic secretion (**Figure 5G**) was met with a massive compensatory upregulation following the intervention, fully restoring the enzymatic transcript pool, including essential lipases (*Pnlip*) and amylases (*Amy2a1*) (**Figure 5H**).

Collectively, these transcriptomic data confirm that the pathological pooling of endogenous FXR antagonists in the aged gut does not merely trigger isolated cellular senescence, but imposes a dominant, systemic suppressive tone that silences tissue-specific functional identities. By depleting this toxic systemic pool, we successfully uncoupled chronological age from transcriptional decline, achieving a profound, multi-organ transcriptomic change that functionally links the intestinal metabolome to systemic structural and metabolic homeostasis.

## 4 Discussion

The aging process is intricately linked to a progressive decline in systemic metabolic homeostasis, yet the mechanisms by which localized microbial-host co-metabolism drives widespread tissue deterioration remain incompletely understood. Élie Metchnikoff proposed over a century ago that aging began in the gut due to “poisonous substances,” so-called “autotoxins,” produced by “putrefactive” bacteria residing in the colon (30). In this study, we unveil a novel, spatiotemporal paradigm of aging driven by the profound dysregulation of the enterohepatic bile acid (BA) network. We demonstrate that advancing age fundamentally reorganizes the systemic BA metabolome, transforming the intestinal lumen into a pathological modulator for endogenous Farnesoid X Receptor (FXR) antagonists. This localized pooling progressively changes the peripheral barrier and immune tissues by altering the levels of critical regulatory molecules. The alteration precipitating a systemic FXR overreaction that drives structural decline, cellular senescence, and immune evasion. Crucially, we show that interrupting this pathological BA signaling axis via systemic sequestration is sufficient to orchestrate a multi-organ reversal of age-associated changes, including transcriptomic and phenotypic changes.

The cornerstone of our model is the severe compartmentalized dysregulation of the BA pool observed in aged mice. While youthful physiology maintains high circulating levels of potent conjugated FXR antagonists, specifically Tα-MCA and Tω-MCA (14, 31), advancing age severely restricts their biodistribution. The progressive accumulation of these specific antagonists within the intestinal lumen—concurrent with their near-total depletion in the mLN, lung, and skin—suggests an age-associated failure in active BA reabsorption. This “intestinal modulation” effectively serves the metabolic communication between the gut and the periphery. Consequently, peripheral tissues are subjected to an inverted agonist-to-antagonist ratio: they are stripped of their endogenous FXR inhibitors just as an age-associated influx of potent, microbial-derived secondary BA agonists, such as DCA and LCA, inundates them.

We identify this resulting peripheral FXR hyperactivation as a primary upstream driver of localized tissue aging. In the skin, this pathological FXR overreaction directly impaired the Retinoic Acid Receptor alpha (RARα) signaling axis, a critical pathway for epithelial homeostasis and differentiation. Aging induced a massive, dysfunctional accumulation of total RARα protein while severely suppressing its active, phosphorylated state (ser77). By using cholestyramine to deplete the systemic BA pool, we successfully normalized FXR expression and restored the balance of functional RARα phosphorylation. This molecular rescue underscores the intense crosstalk between nuclear receptor networks and highlights how the gut-derived metabolome dictates the tone of peripheral receptor signaling.

Beyond receptor crosstalk, our data functionally link the aged BA pool to the establishment and maintenance of cellular senescence. The age-induced FXR overreaction strongly correlated with the pathological accumulation of core senescence regulators—P16, P21, and P53—across multiple peripheral tissues, including the skin and white adipose depots. Notably, systemic BA depletion not only cleared these senescent markers but also actively suppressed the expression of immune checkpoint molecules (PD-L1 and PD-L2) on the surfaces of remaining senescent cells in critical immune and barrier sites, such as the lung, liver, and mLN. This is a critical mechanistic finding; it suggests that the dysregulated aged BA pool does not merely accelerate the transition to senescence but actively shields senescent cells from immune-mediated clearance by maintaining a highly immunosuppressive, PD-L1/PD-L2-rich microenvironment. By stripping this metabolic shield, BA sequestration likely restores immune surveillance, facilitating the clearance of the senescent burden.

Our multi-organ transcriptomic profiling definitively captured the profound systemic impact of this metabolic intervention. Advancing age imposed a dominant, systemic transcriptional suppression that silenced foundational tissue identities—from the collapse of structural collagens (32) and elastins (33) in the lung and keratins (34, 35) in the skin, to the shutdown of primary metabolic hubs (AMPK (36), PPAR (37, 38), CYP450) in the mLN and spleen.

The ability of a simple, gut-restricted intervention like cholestyramine to globally reverse this transcriptomic decline and reactivate these specific structural and metabolic networks is striking. It confirms that the age-related breakdown of tissue architecture is not an irreversible, programmed decay, but rather a continuous physiological response to a toxic systemic signaling environment.

In conclusion, our findings reframe the aging enterohepatic axis not simply as a passive victim of age-related microbiome drift, but as an active, targetable engine of systemic aging. The localized intestinal pooling of FXR antagonists and the subsequent peripheral FXR hyperactivation represent a fundamental breakdown in systemic metabolic communication.

However, we acknowledge that FXR activity was inferred from protein expression levels rather than direct transcriptional activity assays. Future studies employing FXR luciferase reporter mice or chromatin immunoprecipitation would be required to establish FXR hyperactivation in aged peripheral tissues definitively.

An additional limitation is the use of cholestyramine as a BA sequestrant. While effective at depleting the systemic BA pool, cholestyramine has broad effects beyond BA binding, including potential interactions with other luminal contents and indirect effects on gut microbiota composition. Thus, the observed phenotypic improvements cannot be definitively attributed solely to BA depletion without confirmatory studies using more specific approaches, such as intestine-specific FXR antagonists or genetic ablation of BA transporters. Furthermore, the relatively small cohort sizes may limit statistical power and generalizability, and therefore, the findings should be interpreted cautiously until validated in larger independent studies.

By demonstrating that interventions targeting the BA pool can simultaneously restore nuclear receptor balance, clear senescent cells, break immune evasion, and orchestrate transcriptomic changes across multiple organ systems, this study provides a compelling rationale for developing BA-modulating therapies—such as targeted sequestrants or localized FXR modulators—as broad-spectrum geroprotective strategies.

## Data Availability

The raw data supporting the conclusions of this article will be made available by the authors, without undue reservation.

## References

[1] López-Otín C Blasco MA Partridge L Serrano M Kroemer G . The hallmarks of aging. Cell. (2013) 153:1194–217. doi: 10.1016/j.cell.2013.05.039 PMC3836174 23746838

[2] Muñoz-Espín D Serrano M . Cellular senescence: from physiology to pathology. Nat Rev Mol Cell Biol. (2014) 15:482–96. doi: 10.1038/nrm3823 24954210

[3] Kirkwood TB . Understanding the odd science of aging. Cell. (2005) 120:437–47. doi: 10.1016/j.cell.2005.01.027 15734677

[4] He S Sharpless NE . Senescence in health and disease. Cell. (2017) 169:1000–11. doi: 10.1016/j.cell.2017.05.015 28575665 PMC5643029

[5] Wang B Han J Elisseeff JH Demaria M . The senescence-associated secretory phenotype and its physiological and pathological implications. Nat Rev Mol Cell Biol. (2024) 25:958–78. doi: 10.1038/s41580-024-00727-x 38654098

[6] Birch J Gil J . Senescence and the SASP: many therapeutic avenues. Genes Dev. (2020) 34:1565–76. doi: 10.1101/gad.343129.120 33262144 PMC7706700

[7] Aubert G Lansdorp PM . Telomeres and aging. Physiol Rev. (2008) 88:557–79. doi: 10.1152/physrev.00026.2007 18391173

[8] Sato Y Atarashi K Plichta DR Arai Y Sasajima S Kearney SM . Novel bile acid biosynthetic pathways are enriched in the microbiome of centenarians. Nature. (2021) 599:458–64. doi: 10.1038/s41586-021-03832-5 34325466

[9] de Aguiar Vallim TQ Tarling EJ Edwards PA . Pleiotropic roles of bile acids in metabolism. Cell Metab. (2013) 17:657–69. doi: 10.1016/j.cmet.2013.03.013 23602448 PMC3654004

[10] Lefebvre P Cariou B Lien F Kuipers F Staels B . Role of bile acids and bile acid receptors in metabolic regulation. Physiol Rev. (2009) 89:147–91. doi: 10.1152/physrev.00010.2008 19126757

[11] Postler TS Ghosh S . Understanding the holobiont: How microbial metabolites affect human health and shape the immune system. Cell Metab. (2017) 26:110–30. doi: 10.1016/j.cmet.2017.05.008 28625867 PMC5535818

[12] Jia W Xie G Jia W . Bile acid-microbiota crosstalk in gastrointestinal inflammation and carcinogenesis. Nat Rev Gastroenterol Hepatol. (2018) 15:111–28. doi: 10.1038/nrgastro.2017.119 29018272 PMC5899973

[13] Bárcena C Valdés-Mas R Mayoral P Garabaya C Durand S Rodríguez F . Healthspan and lifespan extension by fecal microbiota transplantation into progeroid mice. Nat Med. (2019) 25:1234–42. doi: 10.1038/s41591-019-0504-5 31332389

[14] Sayin SI Wahlstrom A Felin J Jantti S Marschall HU Bamberg K . Gut microbiota regulates bile acid metabolism by reducing the levels of tauro-beta-muricholic acid, a naturally occurring FXR antagonist. Cell Metab. (2013) 17:225–35. doi: 10.1016/j.cmet.2013.01.003 23395169

[15] Wu R Yuan X Li X Ma N Jiang H Tang H . The bile acid-activated retinoic acid response in dendritic cells is involved in food allergen sensitization. Allergy. (2022) 77:483–98. doi: 10.1111/all.15039 34365653

[16] Jiang H Xiong W Feng Y Li X Tan J Zhang Z . Bile acids enrichment fuel tumor aerobic glycolysis and immune evasion via stabilizing FXR-RARalpha. Front Immunol. (2026) 17:1750358. doi: 10.3389/fimmu.2026.1750358 41958659 PMC13057493

[17] Katerndahl CDS Rogers ORS Day RB Xu Z Helton NM Ramakrishnan SM . PML::RARA and GATA2 proteins interact via DNA templates to induce aberrant self-renewal in mouse and human hematopoietic cells. Proc Natl Acad Sci USA. (2024) 121:e2317690121. doi: 10.1073/pnas.2317690121 38648485 PMC11067031

[18] Li X Tan J Xiong W Feng Y Zhang Z . Silica-induced ferroptosis activates retinoic acid signaling in dendritic cells to drive inflammation and fibrosis in silicosis. Int Immunopharmacol. (2025) 149:114244. doi: 10.1016/j.intimp.2025.114244 39938311

[19] Erhuma A Salter AM Sculley DV Langley-Evans SC Bennett AJ . Prenatal exposure to a low-protein diet programs disordered regulation of lipid metabolism in the aging rat. Am J Physiol Endocrinol Metab. (2007) 292:E1702–14. doi: 10.1152/ajpendo.00605.2006 17299084 PMC1890310

[20] Vilà L Roglans N Alegret M Camins A Pallàs M Sánchez RM . Hypertriglyceridemia and hepatic steatosis in senescence-accelerated mouse associate to changes in lipid-related gene expression. J Gerontol A Biol Sci Med Sci. (2007) 62:1219–27. doi: 10.1093/gerona/62.11.1219 18000141

[21] Shilkaitis A Green A Christov K . Retinoids induce cellular senescence in breast cancer cells by RAR-β dependent and independent pathways: Potential clinical implications (Review). Int J Oncol. (2015) 47:35–42. doi: 10.3892/ijo.2015.3013 25997921 PMC4485653

[22] Watson RE Arjuna Ratnayaka J Brooke RC Yee-Sit-Yu S Ancian P Griffiths CE . Retinoic acid receptor alpha expression and cutaneous ageing. Mech Ageing Dev. (2004) 125:465–73. doi: 10.1016/s0047-6374(04)00088-0 15246741

[23] Campisi J Kapahi P Lithgow GJ Melov S Newman JC Verdin E . From discoveries in ageing research to therapeutics for healthy ageing. Nature. (2019) 571:183–92. doi: 10.1038/s41586-019-1365-2 31292558 PMC7205183

[24] Milanovic M Fan DNY Belenki D Däbritz JHM Zhao Z Yu Y . Senescence-associated reprogramming promotes cancer stemness. Nature. (2018) 553:96–100. doi: 10.1038/nature25167 29258294

[25] Suda M Shimizu I Katsuumi G Yoshida Y Hayashi Y Ikegami R . Senolytic vaccination improves normal and pathological age-related phenotypes and increases lifespan in progeroid mice. Nat Aging. (2021) 1:1117–26. doi: 10.1038/s43587-021-00151-2 37117524

[26] Baker DJ Childs BG Durik M Wijers ME Sieben CJ Zhong J . Naturally occurring p16(Ink4a)-positive cells shorten healthy lifespan. Nature. (2016) 530:184–9. doi: 10.1038/nature16932 26840489 PMC4845101

[27] Wang TW Johmura Y Suzuki N Omori S Migita T Yamaguchi K . Blocking PD-L1-PD-1 improves senescence surveillance and ageing phenotypes. Nature. (2022) 611:358–64. doi: 10.1038/s41586-022-05388-4 36323784

[28] Latchman Y Wood CR Chernova T Chaudhary D Borde M Chernova I . PD-L2 is a second ligand for PD-1 and inhibits T cell activation. Nat Immunol. (2001) 2:261–8. doi: 10.1038/85330 11224527

[29] Dimri GP Lee X Basile G Acosta M Scott G Roskelley C . A biomarker that identifies senescent human cells in culture and in aging skin *in vivo*. Proc Natl Acad Sci USA. (1995) 92:9363–7. doi: 10.1073/pnas.92.20.9363 7568133 PMC40985

[30] Metchnikoff E Mitchell PC . The prolongation of life :optimistic studies. (1908), 1. v–xx.

[31] Yang Y Hao C Jiao T Yang Z Li H Zhang Y . Osteoarthritis treatment via the GLP-1-mediated gut-joint axis targets intestinal FXR signaling. Science. (2025) 388:eadt0548. doi: 10.1126/science.adt0548 40179178

[32] Last JA Reiser KM . Collagen biosynthesis. Environ Health Perspect. (1984) 55:169–77. doi: 10.2307/3429701 PMC1568368 6428877

[33] Vindin HJ Oliver BG Weiss AS . Elastin in healthy and diseased lung. Curr Opin Biotechnol. (2022) 74:15–20. doi: 10.1016/j.copbio.2021.10.025 34781101

[34] Omary MB Coulombe PA McLean WH . Intermediate filament proteins and their associated diseases. N Engl J Med. (2004) 351:2087–100. doi: 10.1056/nejmra040319 15537907

[35] Coulombe PA Wong P . Cytoplasmic intermediate filaments revealed as dynamic and multipurpose scaffolds. Nat Cell Biol. (2004) 6:699–706. doi: 10.1038/ncb0804-699 15303099

[36] Burkewitz K Zhang Y Mair WB . AMPK at the nexus of energetics and aging. Cell Metab. (2014) 20:10–25. doi: 10.1016/j.cmet.2014.03.002 24726383 PMC4287273

[37] Zhang R Zheng F . PPAR-gamma and aging: one link through klotho? Kidney Int. (2008) 74:702–4. doi: 10.1038/ki.2008.382 18756295

[38] Zhang H Li Y Fan Y Wu J Zhao B Guan Y . Klotho is a target gene of PPAR-gamma. Kidney Int. (2008) 74:732–9. doi: 10.1038/ki.2008.244 18547997

