# Supplementary material for: Intestinal pooling of taurine conjugated bile acids is associated with tissue aging and senescent immune evasion
Source: Front Immunol. 2026 Jul 28;17:1845117. doi: 10.3389/fimmu.2026.1845117 (PMC13457052; doi:10.3389/fimmu.2026.1845117)
Supplement: Supplementary file 1 [file DataSheet2.pdf]

frontiers

The International Society for the Advancement of Cytometry (ISAC) has highlighted the importance of including comprehensive methodological information to ensure data reproducibility and reliability. In line with this, Frontiers in Immunology now requires authors to submit a checklist for manuscripts that involve flow or mass cytometry. This checklist helps standardize the reporting process, improving the quality and transparency of published data. By doing so, we support scientific progress, making it easier for other researchers to replicate and validate experiments.

This form should be submitted with any manuscripts using flow or mass cytometry.

Sample/specimen/material description

☐ Total blood

☑ PBMCs

☐ Organ digests

Other____

Did the samples suffer any treatment before or after incubation with the antibodies?

☐ Drug ____________________________________

☐ Cell permeabilization ______________________

☐ Dye _____________________________________

☐ Propidium iodine

☑ Not applicable

Other ______________________________________

Instrument and antibodies

Name of the Cytometer ACEA Novocyte

Antibodies and targets	Fluorochrome/ Metal	Catalog number/Company
e.g. anti-CD4	FITC	Cat. XXX/ XXX ltd.
SPiDER-β-Gal	FITC	Cat.SG03/DOJINDO

PD-L1 (B7-H1)	PE	Cat.124307/Biolegend
PD-L2 (B7-DC)	PE/DazzleTM 594	Cat.107215/Biolegend

Data analyses

1. Name of the software FlowJo_v10.8.1
2. Reference gating strategy in the manuscript or supplementary material

Gating strategy in (eg Figure X) 1.Initial gating was performed on FSC-A vs. SSC-A to exclude debris
2. The gating hierarchy was established as follows: SPiDER-β -gal +cells → PDL1+ cells /PDL2+ cells. (eg Figure 4A)
